# Tumors of the Brain Disclosed by Autopsy

**Published:** 1895-08

**Authors:** H. D. Gill


### Tumors of the Brain Disclosed by Autopsy.

BY H. D. GILL, V.S.

"Lydtin," quoted by "Dieckerhoff," has given the history of a twenty-year-old horse which for ten years had shown complete atrophy of the masseter and temporal muscles on the right side. No other symptoms presented themselves till, without warning, a "comatose condition" developed; the patient took no food or drink, and could not swallow. After a few days he was killed, and at the autopsy a tough nodular tumor half the size of the cerebellum was found over the petrous bone. The tumor enveloped the trigeminal nerve and extended forward to the right hemisphere, and behind to the medulla. It was found to be a fibro-sarcoma which had risen in the dura.

Dr. Samuel Atchison, of Brooklyn, recently sent to the laboratory a brain of a horse with the following history: The horse, which was not young, had had no symptoms of illness of any sort till one evening a frenzy apparently seized him; there were several almost convulsive attacks, and about the middle of the night the animal died. Examination of the brain showed two tumors, each slightly less in size than a hen's egg, springing respectively from the posterior portions of the right and left choroid plexuses, within the ventricles; each was attached by a narrow pedicle; the surface was tough and fairly dense; within the tissue was spongy and contained considerable clear fluid. Microscopic examination showed the tumor to be a fibro-sarcoma.

